# Spontaneous Recovery of Paraplegia Caused by Spinal Epidural Hematoma after Removal of Epidural Catheter

**DOI:** 10.1155/2014/291728

**Published:** 2014-05-05

**Authors:** Kouhei Iwashita, Kenji Shigematsu, Kazuo Higa, Keiichi Nitahara

**Affiliations:** Department of Anesthesiology, Fukuoka University Faculty of Medicine, 7-45-1 Nanakuma, Jonan-ku, Fukuoka 814-0180, Japan

## Abstract

We report a patient who developed paraplegia caused by a spinal epidural hematoma after removal of an epidural catheter, which resolved spontaneously. A 60-year-old woman underwent thoracoscopic partial resection of the left lung under general anesthesia combined with epidural anesthesia. She neither was coagulopathic nor had received anticoagulants. Paraplegia occurred 40 minutes after removal of the epidural catheter on the first postoperative day. Magnetic resonance images revealed a spinal epidural hematoma. Surgery was not required as the paraplegia gradually improved until, within 1 hour, it had completely resolved. Hypoesthesia had completely resolved by the third postoperative day.

## 1. Introduction

Spinal epidural hematoma (SEH) associated with epidural anesthesia is very rare; however, it is a serious complication. It is recommended that surgery is undertaken as quickly as possible to avoid permanent neurologic deficits [1]. Nonetheless, there have been some reports of spontaneous resolution of neurologic deficits caused by SEH associated with epidural anesthesia [2–10].

We report a patient, with normal clotting function, who had not received anticoagulant therapy and developed paraplegia due to a SEH after removal of an epidural catheter, which spontaneously resolved rapidly and completely.

## 2. Case Report

A 60-year-old, 151 cm, 47 kg woman with a left lung tumor was scheduled for video-assisted thoracoscopic partial lung resection. She had a history of hypertension and type 2 diabetes mellitus and was treated with valsartan and nifedipine. She had normal renal and hepatic function and took no drugs that might be expected to impair blood clotting. Preoperative laboratory investigations showed a blood hemoglobin concentration of 14.3 g/dL, hematocrit of 43%, platelet count of 191 × 10^3^/*μ*L, international normalized ratio of prothrombin time (PT-INR) of 0.92 (reference value: 0.85–1.15), and activated partial thromboplastin time (APTT) of 31.2 s (reference value: 24.0–38.0 s).

She received premedication with oral diazepam 5 mg, pentobarbital 100 mg, and roxatidine 75 mg, 3 h before entering the operating room. Electrocardiography, noninvasive blood pressure, and pulse oximetry were monitored. She was placed in the right lateral decubitus position and the epidural space was identified using a paramedian approach at the T5-T6 interspace with the loss of resistance to physiologic saline technique. An 18-gauge epidural catheter was advanced cephalad with slight resistance, but she did not complain of paresthesia. Neither blood nor cerebrospinal fluid was aspirated through the catheter. She felt pain at the epidural catheter insertion site on injection of a test dose of 2 mL 0.375% ropivacaine, but there was no pain when ropivacaine was given slowly. An additional 6 mL 0.375% ropivacaine was slowly injected into the epidural space 5 minutes after the test dose. The procedure was otherwise uneventful.

General anesthesia was induced with propofol 100 mg. The trachea was intubated after administration of vecuronium 6 mg. Thereafter, anesthesia was maintained with 1-2% sevoflurane. Additional 3 mL 0.375% ropivacaine was injected epidurally at the beginning of the surgery. The intraoperative course was uneventful. The duration of surgery was 70 minutes. Blood loss was minimal, urine output was 350 mL, and 950 mL intravenous fluid was given during the surgery. Postoperative analgesia was provided by means of patient-controlled epidural analgesia (PCEA) using 0.2% ropivacaine with 2 *μ*g/mL fentanyl. It was infused at a rate of 4 mL/h with a demand bolus injection of 3 mL. The lockout time was 30 minutes. No anticoagulants were given postoperatively.

On the first postoperative day, the patient experienced pain in the right side of the back and the right upper limb during infusion of PCEA demand boluses. There was hypoesthesia over the left chest; however, sensation was normal over the right chest. The epidural catheter was withdrawn by 1 cm, but the pain in the back and the upper limb on bolus injection persisted. The epidural catheter was removed. There was no resistance during removal of the catheter. Manual compression was applied to stop bleeding from the catheter insertion site.

**Figure 1:**
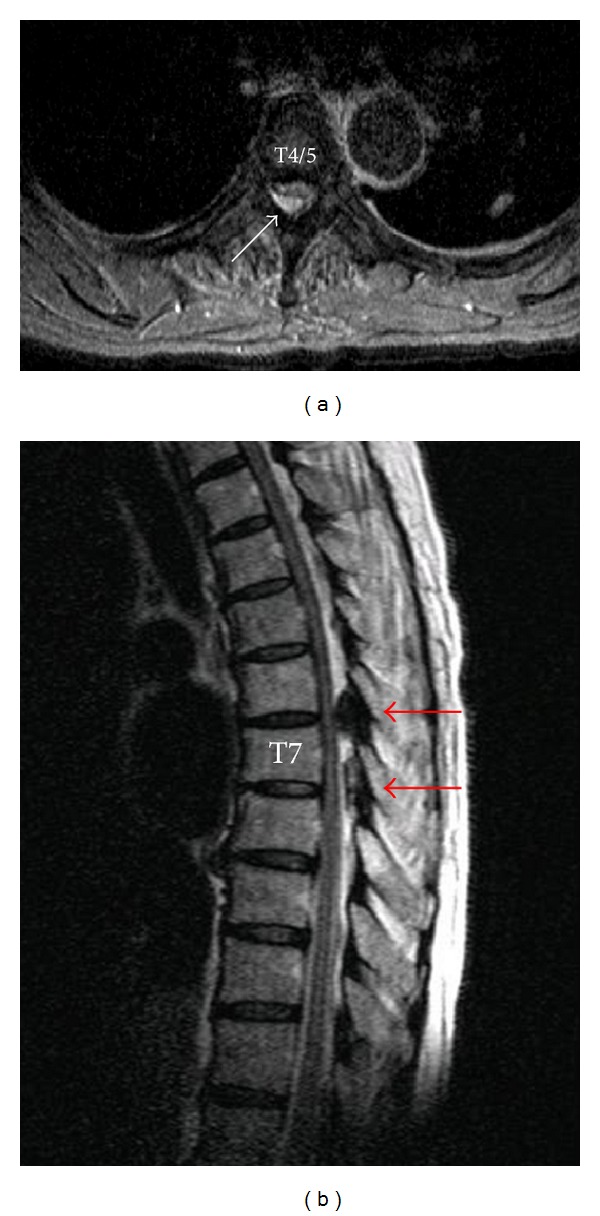
Magnetic resonance images one hour after onset of paraplegia. (a) T2-weighted image. Arrow indicates hematoma compressing the dural sac at T4/5. (b) T2-weighted image. Arrows indicate ossification of the posterior longitudinal ligament at T6/7 and T7/8.

She vomited 10 minutes after removal of the epidural catheter. Progressive paralysis of the lower limbs occurred immediately afterwards and complete paraplegia had developed within 40 minutes. On physical examination, there was bilateral hypoesthesia below the T5 dermatome and the anal sphincter reflex was impaired. Magnetic resonance (MR) images taken 1 h after removal of the epidural catheter revealed a hematoma in the dorsal epidural space extending from T1 to T9, which compressed the dural sac from T4 to T5 (Figure 1(a)). The MR images also revealed ossification of the posterior longitudinal ligament (OPLL) at T6/7 and T7/8, but this did not compress the dural sac (Figure 1(b)). Paralysis and hypoesthesia began to improve gradually, even while the MR images were being acquired. Surgical intervention was not needed as the sensorimotor deficits continued to show steady improvement. The motor deficits had resolved completely within 2 h of removal of the epidural catheter. Slight hypoesthesia persisted on the medial aspect of the right leg. Laboratory investigations showed a platelet count of 158 × 10^3^/*μ*L, PT-INR of 1.03, and APTT of 27.4 s. The sensory deficits in the right leg resolved completely by the third day after surgery and she was able to walk by the fifth postoperative day. The left lung nodule proved to be an epithelioid granuloma. No hematoma was visible in the epidural space on MR images acquired on the twelfth day after surgery. She was discharged without neurologic deficit.

The Institutional Review Board of Fukuoka University Hospital gave permission for this report to be published (number 13-6-17).

## 3. Discussion

The incidence of SEH associated with epidural anesthesia is estimated to be between 1 : 150,000 and 1 : 190,000 [11, 12]. Risk factors for SEH associated with epidural anesthesia are female sex, old age, a history of gastrointestinal bleeding, anticoagulant therapy, coagulopathy, hepatic dysfunction, and difficult or traumatic epidural catheterization and bleeding at the puncture site [2, 5, 13]. Even if bleeding occurs in the epidural space, it usually stops rapidly [13]; however, a large hematoma may accumulate in the epidural space of patients with coagulopathy or liver dysfunction and those taking anticoagulants [13]. Spinal epidural hematoma may occur in patients with normal clotting function [5, 13, 14]. It may present when an epidural catheter is* in situ* or after its removal [2–10]. In this case, the patient vomited after removal of the epidural catheter, and thereafter paraplegia occurred rapidly. The MR images acquired immediately after the onset of paraplegia showed SEH at the epidural catheter insertion site, and consequently we diagnosed paraplegia due to an epidural catheter-related SEH. The patient had normal clotting function and was not taking anticoagulants and insertion was uneventful, suggesting that the hematoma occurred after removal of the epidural catheter.

Paraplegia is reported to have occurred due to compression of the spinal cord by local anesthetic that had pooled in the epidural space in an elderly patient with kyphosis after an epidural catheter had been placed [15]. In that case, the quality of epidural analgesia was reportedly good, and paralysis of the lower limbs became evident on the second postoperative day. Paralysis resolved once the epidural catheter had been removed. In our case, epidural analgesia was unilateral and inadequate on one side, and paraplegia progressed rapidly after removal of the epidural catheter, so it seems unlikely that paralysis could have occurred as a result of pooling of local anesthetic.

A small volume of epidural clot is reported to have caused spinal cord compression and paraplegia that resolved spontaneously in a patient with thoracic spinal stenosis [6]. In this case, the patient had previously undiagnosed ossification of the posterior longitudinal ligament at T6/7 and T7/8. This may have caused pooling of blood at the site of catheter insertion (T5/6) when the catheter was removed. A degree of local tamponade may have assisted hemostasis, subsequent craniocaudal diffusion of the hematoma decompressed the cord, and paralysis resolved spontaneously within 2 h.

After diagnosis of SEH, early surgical intervention has been strongly recommended to avoid permanent neurologic deficits: patients who undergo surgical spinal decompression less than 12 h after the onset of symptoms or signs have a superior outcome [1, 12]. Nonetheless, we found nine reports of patients with SEH associated with epidural anesthesia whose symptoms recovered spontaneously [2–10]. Three of these cases are not discussed herein, as they did not develop paraplegia [8–10]. The details of the remaining seven patients (cases 2–7 presented alongside our patient) are shown in Table 1.

Preoperative coagulation studies were within the normal ranges in six of the seven patients (cases 2–6 and our patient) [2–6]. One patient (case 7) had preoperative coagulopathy due to cirrhosis of the liver [7]. Epidural puncture was straightforward in three patients (cases 5 and 6 and ours) [5, 6]. There was backflow of blood through the Tuohy needle after epidural puncture in one patient and the catheter was subsequently placed at a different level (case 7) [7]. Three patients (cases 2–4) had received postoperative anticoagulants [2–4]: two (cases 2 and 4) received low-molecular-weight heparin [2, 4] and one (case 3) unfractionated heparin [3]. When SEH occurred, coagulation studies and platelet counts were within their normal ranges in four patients (cases 2, 3, and 5 and ours) [2, 3, 5], not described in two patients (cases 4 and 6) [4, 6], while the patient with liver cirrhosis was coagulopathic (case 7) [7]. Thus, for most patients who recovered spontaneously, perioperative hemostasis was apparently normal and the epidural puncture was seemingly straightforward.

Neurologic deficits began to improve within 1 to 24 h after symptom onset and neurologic function recovered completely in six of the seven patients (cases 2–6 and our patient) [2–6]. In the remaining case, neurologic deficits began to improve within 4 days of symptom onset and the patient was left with slight hypoesthesia on the left leg (case 7) [7]. Three patients (cases 4, 5, and 7) received steroid treatment for SEH [4, 5, 7]. Surgery was not performed in five patients because motor function began to return to the lower limbs during assessment and investigation (cases 2 and 4–6 and ours) [2, 4–6], because of the age and poor general condition of one patient (case 3) [3] and because symptoms had stabilized (case 7) [7]. Our patient developed paraplegia 40 minutes after removal of the epidural catheter. Surgery was not performed because motor function began to improve during MR image acquisition and sensorimotor function had almost completely recovered within 2 days of symptom onset. Therefore, if neurologic deficits begin to recover shortly after the onset of paraplegia, it may be possible to avoid surgery.

Here we report a case of paraplegia due to SEH after removal of an epidural catheter that resolved completely without surgery. We have found six reports of similar cases. It is difficult to identify why paraplegia improved spontaneously in these cases; however, early improvement of neurologic deficits may predict spontaneous recovery.

## Figures and Tables

**Table 1a:** (a)

Reference	Sex	Age (year)	Diagnosis, Operation	Concurrent disease	Preoperative PLT counts and coagulation studies	Epidural puncture
Our case	F	60	Epithelioid granuloma, thracoscopic partial lung resection	HT, DM	PLT 191 × 10^3^/*μ*L PT-INR 0.92 APTT 26.7 s	Straightforward
[2]	F	78	Polyarticular rheumatoid arthritis, total knee replacement	HT, DM	WNL	Straightforward
[3]	F	90	Gastric adenocarcinoma, subtotal gastrectomy	Peptic ulcer disease	WNL	Straightforward
[4]	F	73	Kidney tumor, left nephrectomy	None	WNL	Straightforward
[5]	F	72	Bile duct stenosis and cholelithiasis, choledochoduodenostomy and cholecystectomy	None	WNL	Straightforward
[6]	M	71	Gastric cancer and rectal cancer, miles surgery and gastrectomy	HT, ossification of the posterior longitudinal ligament (T10-L1)	WNL	Straightforward
[7]	F	69	Lung cancer, partial resection of the lung	Cirrhosis of the liver	PLT 121 × 10^3^/*μ*L PT 54%	Prior attempt at T7/8 abandoned due to backflow of blood in the Touhy needle

SEH: spinal epidural hematoma, PLT: Platelet count, HT: hypertension, DM: diabetes mellitus, PT: prothrombin time, INR: international normalized ratio, APTT: activated partial thromboplastin time, POD: postoperative day, WNL: within normal limits, EDC: epidural catheter, MRI: magnetic resonance image, NS: not stated.

**Table 1b:** (b)

Reference	Insertion site of epidural catheter	Anticoagulant therapy	Onset of symptoms	Epidural catheter at the time of onset of paraplegia	Symptoms
Our case	T5/6	None	POD 1	10 min after removal	Vomiting Paraplegia
[2]	L2/3	Enoxaparin, postoperatively	POD 2	Indwelling	Back pain Paraplegia
[3]	T8/9	Unfractionated heparin, postoperatively	POD 2	Indwelling	Paraplegia
[4]	T12/L1	Enoxaparin 24 h before surgery until 2 h before removal of EDC on POD 3	POD 3	Immediately after removal	Back pain Paraplegia Absent anal sphincter reflex
[5]	T7/8	None	POD 2	Indwelling	Paraplegia
[6]	T12/L1	None	POD 5	30 min after removal	Paraplegia
[7]	T9/10	None	POD 5	After surgery, blood was noted at the insertion point of the catheter, which was then the removed	Paraplegia

**Table 1c:** (c)

Reference	Extent of SEH	Coagulation studies at the time of SEH occurrence	Time to improvement after occurrence of paraplegia	Treatment	Reason that surgery was not done	Follow-up neurologic deficits
Our case	T1-9	PLT 158 × 10^3^/*μ*L PT-INR 1.03 APTT 27.4 s	1 h	None	Motor function in legs began to return during MRI	3 d later: none
[2]	T10-L1	PLT 170 × 10^3^/*μ*L PT-INR 1.04	On the same day	NS	Motor function in legs began to return before surgery	3 mon later: none
[3]	T3-11	PLT 185 × 10^3^/*μ*L PT-INR 1.1 APTT 32.6 s	By the next morning	NS	Age, advanced malignancy, >12 h after paraplegia occurred	56 d later: none
[4]	T11-L1	NS	On the same day	Dexamethasone	At the time of the neurosurgical examination, motor function had returned to legs	7 d later: none
[5]	T6-9	WNL	On the next day	Dexamethasone	Motor function in legs returned after MRI	40 d later: none
[6]	T11-L1	NS	1 h	None	1 h after paraplegia occurred, paralysis had resolved completely	1 h later: none
[7]	T4-8	PLT 101 × 10^3^/*μ*L PT 42%	Between the next day and POD 4	Glycerol Steroid	On POD 2, neurologic findings had stabilized and seemed unlikely to deteriorate	1 mon later: mild hypoesthesia of left leg
